# Age- and gender-based comorbidity categories in general practitioner and pulmonology patients with COPD

**DOI:** 10.1038/s41533-022-00278-8

**Published:** 2022-05-02

**Authors:** Su-Jong Kim-Dorner, Torben Schmidt, Alexander Kuhlmann, Johann-Matthias Graf von der Schulenburg, Tobias Welte, Heidrun Lingner

**Affiliations:** 1 Medical Psychology, Hannover Medical School, Carl-Neuberg-Straße1, 30625 Hannover, Germany; 2 Center for Health Economics Research Hannover (CHERH), Leibniz University Hannover, Otto-Brenner-Straße 7, 30159 Hannover, Germany; 3 Biomedical Research in Endstage and Obstructive Lung Disease Hannover (BREATH); Member of the German Center for Lung Research (DZL), Hannover, Germany; 4 TW Pulmonology, Hannover Medical School, Carl-Neuberg-Straße 1, 30625 Hannover, Germany

**Keywords:** Medical research, Chronic obstructive pulmonary disease, Health occupations, Quality of life

## Abstract

Chronic obstructive pulmonary disease (COPD) is a debilitating medical condition often accompanied by multiple chronic conditions. COPD is more frequent among older adults and affects both genders. The aim of the current cross-sectional survey was to characterize chronic comorbidities stratified by gender and age among patients with COPD under the care of general practitioners (GP) and pulmonologists, using real-world patient data. A total of 7966 COPD patients (women: 45%) with more than 5 years of the observation period in the practice were examined using 60 different Chronic comorbid conditions (CCC) and Elixhauser measures. More than 9 in 10 patients had at least one, and 51.7% had more than three comorbidities. No gender difference was found in the number of comorbidities. However, men had higher Elixhauser-van Walraven index scores than women, and the types of comorbidities differed by gender. An increasing number of comorbidities was seen with aging but the patients in their 30s and 40s also had a high number of comorbidities. Moreover, GP patients had a higher number and a wider array of documented comorbidities than pulmonology patients did. Psychological comorbidities were common in all patients, but particularly among younger patients. These findings around gender- and age-stratified comorbidities under the care of GPs and pulmonologists have implications for the choice of data provenience for decision-making analysis and treatment selection and success.

## Introduction

Chronic obstructive pulmonary disease (COPD), characterized by chronic obstruction of lung airflow that interferes with normal breathing, claims millions of lives each year and is the fourth leading cause of mortality worldwide^1,2^. COPD used to be more common in men; however, because of increased tobacco use and the higher risk of exposure to indoor air pollution among women, the disease now affects both men and women almost equally^2^. Additionally, COPD is more common among the elderly population as COPD symptoms develop slowly and become apparent during middle age^2,3^. Considering one in six people will be over the age of 65 by 2050^4^, the aging of the general population may further increase the burden of COPD.

COPD patients often have a multitude of other chronic conditions that can influence the prognosis of COPD and complicate the disease management^5^. Patients with COPD often die prematurely after suffering for many years due to COPD or its comorbid conditions^1^. These comorbid conditions increase economic burdens by directly increasing medical costs associated with hospitalization and service utilization and indirectly via early retirement or inability to work^6,7^. Recently, multiple studies have further enhanced our understanding of COPD and associated comorbidities^8–12^, and some highlighted the potential underlying pathophysiology of COPD, the mechanism of systemic inflammation^13,14^. These studies of comorbidities are crucial because thoroughly understanding the nature and pattern of comorbidity is the foundation for physicians to provide broad yet appropriately targeted and prioritized treatments to enhance the COPD treatment outcome.

While past comorbidity studies have revealed invaluable information about the association and potential mechanism of comorbidities, thorough documentation of COPD comorbidities using routine real-world data in primary care settings is still scarce. Moreover, previous studies either have examined a small number of chronic conditions or did not examine gender differences^8–12^. Therefore, our study aimed to examine the comorbidities stratified by gender and age among primary care patients with COPD using routine care data from the electronic patient records of general practice and pulmonology settings. Our goal is to contribute to the creation of a road map of personalized holistic treatment choices, by outlining the gender- and age-specific comorbidities among patients with COPD.

## Methods

### Study design

The current study was a cross-sectional survey using primary care data of the German BeoNet Register-Database (BNR). The BNR is a compilation of all routinely documented information from primary care electronic patient records from general practitioners (GPs) and pulmonologists participating in the network. It comprises a retrospective dataset since the practice used electronic files and the database is updated weekly. Only completely anonymized data are available for research purposes. In Germany, all physicians in outpatient practices are considered primary care physicians because patients have direct access to any physician without a referral and regardless of their specialty^15^. Therefore, the patient records from both GP and pulmonology practices were included in the study. Written informed consent from patients was not required, as the analyses were performed on a de-identified dataset out of the Registry database. The study was approved by the Medizinische Hochschule Hannover (MHH) ethics committee.

### Study patients

Female and male patients aged 20 years and older with physician-diagnosed COPD were included in the analyses. Physician-diagnosed COPD was identified by having two documented diagnostic codes of J44 following the International Statistical Classification of Diseases (ICD-10)^16,17^, and one of which was a permanent diagnosis of COPD. Moreover, patients must have been under observation at least for 5 years, so that the cumulated comorbidities reflect their overall health status. Exclusion criteria were age <20 years old, and/or missing data on major variables such as gender and age. The detailed patient selection process is presented as a flowchart in the Supplementary Document (Supplementary Fig. 1). The patients were stratified into seven age groups based on their age at their last visit: 20s, 30s, 40s, 50s, 60s, 70s, and 80s+ group. Overall comorbidity is stratified and examined by gender and age group.

#### The 10-year index period analysis

We examined comorbidity occurrence during a 10-year index period, 5 years before and 5 years after the index date. The index date was defined as the time of the first office visit with documented COPD. Based on the age of the patients at the index date, three groups were formed and examined: the <45, 45–64, and ≥65 years of index age groups. For this analysis, the patients who had not been with the practice for more than 10 years (5 years before and after the index date) were excluded.

### Comorbidity measures

All ICD codes of each patient entered in the database were examined. Sixty chronic comorbid conditions (CCC) were created by searching and re-coding individual ICD-10 codes into different disease categories corresponding to the classification of Calderón-Larrañaga et al.^18^. Disease category names and codes were retained without any alterations according to the original publication. However, for the purposes of this study, ICD-10 code J44 was excluded from the category named “COPD, emphysema, chronic bronchitis.” The total number of CCC and frequency of 60 categories were examined. In addition, 30 Elixhauser comorbid conditions were generated^19,20^. Although Elixhauser comorbidity includes a smaller number of comorbidity than CCC, the Elixhauser measure was included for its summation scores and index scores based on the van Walraven (vW) algorithm. Elixhauser-vW index scores have been linked to mortality rates and provide additional information, which cannot be assessed using summation scores of each disease category alone^21–27^. Elixhauser-vW index scores were calculated by weighting individual comorbidity categories^21^. The J44 diagnosis was excluded from the total number of comorbidity but not from the Elixhauser-vW index score to adhere to the original algorithm. For the 10-year index period analyses, only the first documentation of each comorbidity was considered.

### Data and statistical analyses

The BNR-database was accessed in September 2020. All available medical records of patients with permanent COPD diagnoses were extracted along with patient demographic information and the documented dates of their practice visits. In the first data processing step, the formats were standardized. Subsequently, the variables needed for the inclusion and exclusion criteria were created. Finally, the diagnosis data were reduced to the ICD codes required by the employed methods. The software R (R Core Team, 2020) was used for this process.

Descriptive statistics are presented as means and standard deviations (SD) or absolute numbers and percentages. Given the robustness of parametric tests with large sample sizes, independent *t*-tests were used to compare demographic gender differences (*χ*^2^ test for categorical variables) and analysis of variance (ANOVA) was used to compare the group differences. The Bonferroni post hoc tests were conducted to follow-up on age group differences. The statistical significance level was set at *p* < 0.05 (two-tailed), and all data were analyzed using the SPSS statistical software package (SPSS, version 26); SPSS and Excel (Microsoft Office Professional Plus 2016) were utilized to create figures.

### Reporting summary

Further information on research design is available in the Nature Research Reporting Summary linked to this article.

## Results

### Sample characteristics

A total of 7966 patients with COPD from 20 GPs and six pulmonologists were included in the study. The 20s age group was small (female = 6; male = 3) and thus excluded from further analyses. Men had a longer observation period in the practice after the first documentation of COPD compared to women (*p* < 0.01). However, the total observation years in practice did not differ by gender. There were more men in the 70s group than women (*p* < 0.05), but no other age groups differed by gender. See Table 1.

**Table 1:** Characteristics of 7957 COPD patients by gender.

Variable	*n* (%) or mean (SD)		
	Female (*n* = 3573, 44.9%)	Male (*n* = 4384, 55.1%)	*p*
Age during the last visit (years)	70.1 (11.9)	70.5 (11.3)	0.12
Age group			<0.05
30s	34 (1.0%)	40 (0.9%)	
40s	145 (4.1%)	138 (3.1%)	
50s	493 (13.8%)	584 (13.3%)	
60s	993 (27.8%)	1155 (26.3%)	
70s	1048 (29.3%)	1430 (32.6%)	
80s+	860 (24.1%)	1037 (23.7%)	
GP (%)/pulmonologist (%)	1021 (28.5%)/2558 (71.5%)	1172 (26.7%)/3215 (73.3%)	0.07
Age of 1st COPD documentation	61.2 (12.7)	61.2 (11.8)	0.99
Duration in practice since 1st COPD documentation (years)	8.9 (6.4)	9.3 (6.5)	<0.01
Observation years in practice	13.9 (6.7)	13.7 (6.6)	0.31

*P* values are based on an independent *t*-test or a *χ*^2^ test.

### Comorbidity by gender and age group

**Fig. 1:**
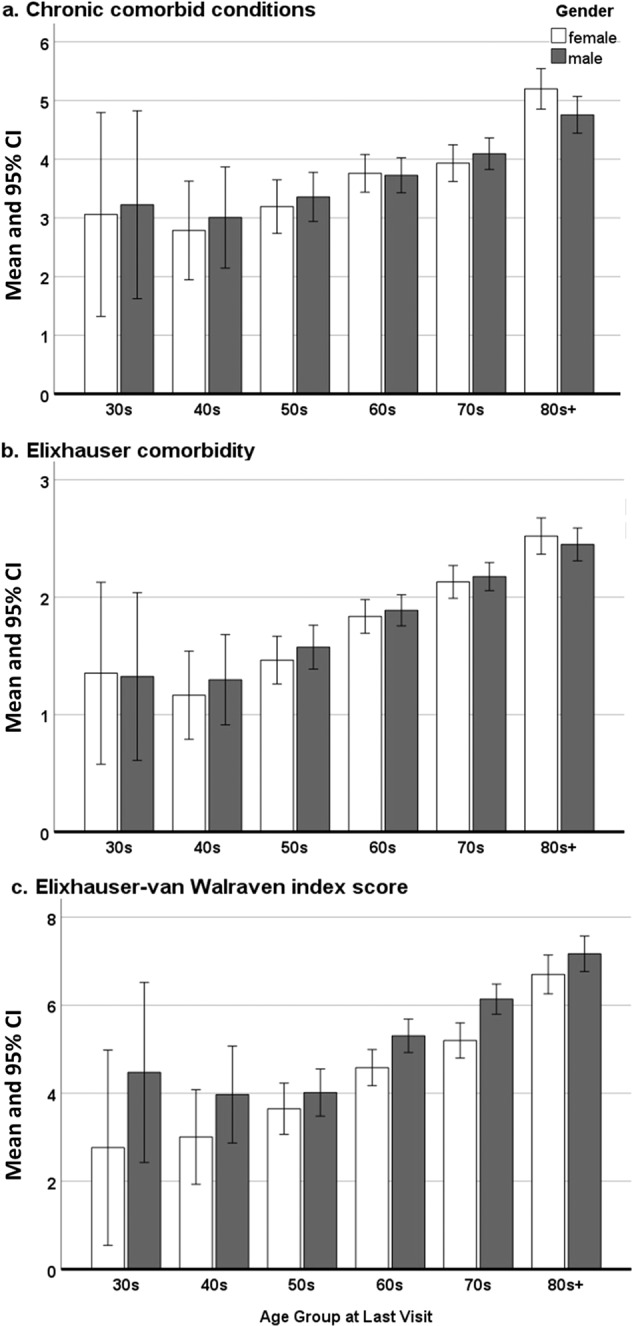
Chronic comorbid conditions (CCC), Elixhauser, and Elixhauser-van Walraven index scores by gender and age group. Mean number of comorbidities with error bars representing 95% confidence intervals.

Comorbidity measures were analyzed using separate 2 × 6 factorial ANOVAs (Gender × Age group). Women and men did not differ in the mean number (M(SD)) of comorbidities assessed by chronic comorbid conditions (CCC) (women: 4.03 (5.40) and men: 4.01 (5.03), *p* = 0.87) or Elixhauser (women: 1.98 (2.31) and men: 2.04 (2.30), *p* = 0.67). In general, older age groups had more comorbidities than the younger groups in both comorbidity measures (both *p* < 0.001). Moreover, this age difference in the prevalence was seen in both genders (all significant at *p* < 0.001) (see Fig. 1a, b). The Bonferroni post hoc tests of age group comparisons showed that for CCC, the 80s group had significantly more comorbidities than all other age groups, and furthermore the 70s group was significantly different from the 40s and 50s groups. For Elixhauser comorbidities, the 80s and 70s groups had a significantly higher number of comorbidities than their respective younger groups, while the 60s group differed significantly from the 40s and the 50s group. For both CCC and Elixhauser, the 30s, 40s, and 50s groups did not significantly differ from each other. No interaction term existed between gender and age. The *p* values from Bonferroni tests are provided in the supplementary document (Supplementary Table 1).

Elixhauser-van Walraven (vW) index scores showed significant effects of gender (*p* < 0.01) and age group (*p* < 0.001). Despite having a similar number of Elixhauser conditions, men had significantly higher mean Elixhauser-vW index scores than women (men: 5.80 (6.97) and women: 5.06 (6.32), *p* < 0.01) (see Fig. 1c). A sensitivity analysis of Elixhauser-vW index scores, excluding J44 diagnosis, also confirmed the finding of the primary analysis that men had significantly higher mean Elixhauser-vW index scores than women (men: 4.33 (7.25) and women: 3.73 (6.55), *p* < 0.05). According to the Bonferroni test of the Elixhauser-vW index score, the 80s group had significantly higher scores than all the other age groups; the 70s had significantly higher index scores compared to all the other younger age groups besides the 30s group; and the 60s group had a significantly higher index score compared to the 40s and 50s. Similar to the findings from CCC and Elixhauser scores, the 30s, 40s, and 50s groups did not significantly differ from each other on the Elixhauser-vW index score. There was no significant gender and age group interaction.

Examination of the Elixhauser-vW index algorithm and the weight values showed that women were diagnosed more frequently with deficiency anemia and depression than men, and these conditions have negative weights of −2 and −3, respectively. In contrast, men were diagnosed more frequently than women with the conditions of positive weights for the algorithm: solid tumor without metastasis, cardiac arrhythmias, congestive heart failure, peripheral vascular disorder, and metastatic cancer (weights: 4, 5, 7, 2, and 12, respectively). Men did not have a higher frequency of any conditions that have negative weights compared to women. Detailed data are provided in the Supplementary Document (Supplementary Table 2).

### Number of comorbidities by gender and age group differentiated by practice types

**Fig. 2:**
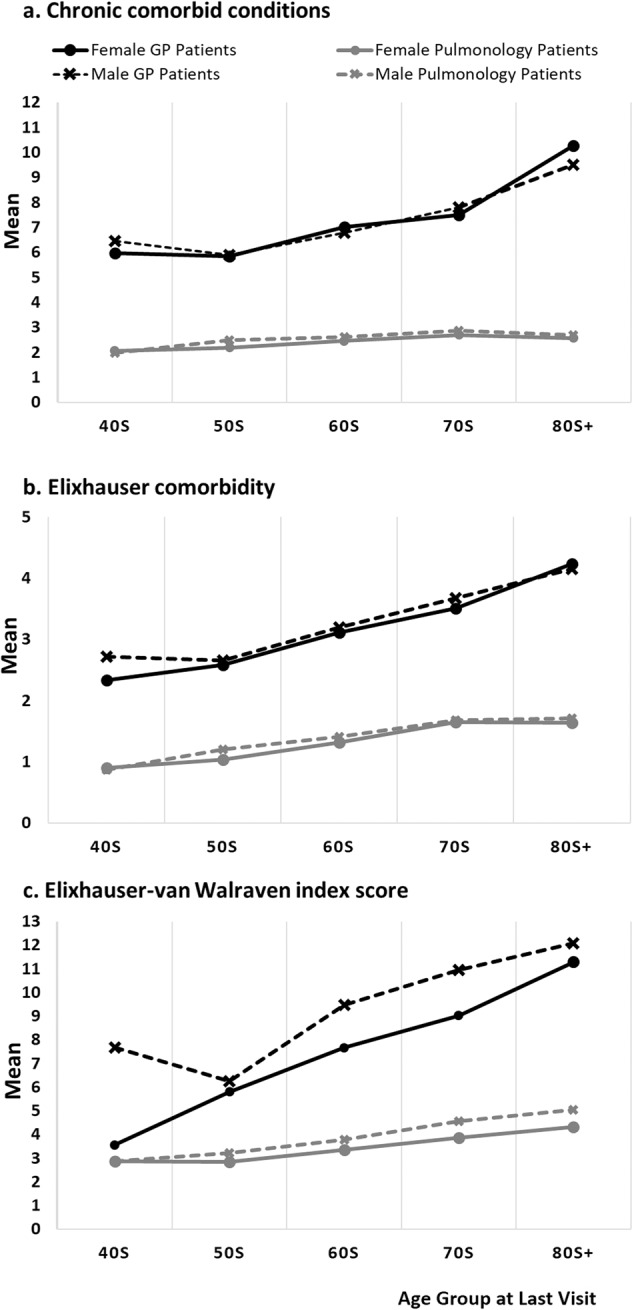
Chronic comorbid conditions (CCC), Elixhauser, and Elixhauser-vW index by gender, age group, and practice type. Figures represent mean number of comorbidities for each age group separated by gender and practice type.

Whether the patient was from a GP or pulmonology practice had a significant impact on the number of comorbidities. A three-way ANOVA test was performed excluding the 30s group due to the small sample size in GP patients (*n* = 13). GP patients had a higher mean number of documented comorbidities than the pulmonology patients for all measures (CCC: 7.8 (8.5) and 2.6 (1.6); Elixhauser: 3.5 (3.7) and 1.5 (1.1), and Elixhauser-vW index score: (9.49 (10.6) and 3.96 (3.2), all significant at *p* < 0.001). Observation years differed by practice types (GP: 14.6 (7.1) vs Pulmonology 13.5 (6.4) years) but accounting for this difference statistically did not change the results. There was no gender difference in both CCC and Elixhauser. However, men had a significantly higher Elixhauser-vW index score than women in both practices (GP: men: 10.2 (11.1) and women: 8.7 (10.0), *p* < 0.01 and Pulmonology: men: 4.2 (3.4) and women: 3.6 (2.9), *p* < 0.001). See Fig. 2 and the Supplementary Document (Supplementary Table 3) for the corresponding data.

As the interaction terms between practice type and age groups were significant, the data were examined by practice type. In both practices, age groups differed in the number of comorbidities in CCC, Elixhauser, and Elixhauser-vW index scores. The Bonferroni post hoc tests of the age group comparisons are provided in the Supplementary Document (Supplementary Table 4).

#### CCC

Among GP patients, the 80s+ group was different from all other age groups. The 70s were only different from the 50s in the sample of GPs patients whereas the 70s in the pulmonologist sample differed from all other younger age groups. For pulmonology patients, the 80s+ group was different from the 40s, 50s, and 70s groups. The 40s group was different from all other groups but not from the 50s (see Fig. 2a).

#### Elixhauser

Among GP patients, the 80s+ group was different from all other age groups except the 70s group, and the 70s group was in addition only different from the 50s groups. For pulmonology patients, all age groups were distinctly different from each other except for the 80s+ to 70s.

#### Elixhauser-vW index score

Among GP patients, the 80s+ was different from all other groups except the 70s. The 70s group was also different from the 40s and 50s, and the 60s only differed from the 50s. Among pulmonology patients, the 80s and 70s, and 60s groups had higher index scores compared to all of their respective younger age groups.

### Percent distribution of patients with comorbidities by gender and age group differentiated by practice types

**Fig. 3:**
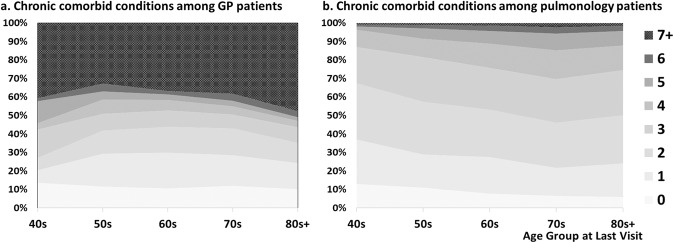
Percent distribution of patients with a number of chronic comorbid conditions (CCC). Figures represent frequency of patients with a number of comorbidities, from 0 to 7 or more, for each age group separated by practice type.

From the entire COPD patient sample, only 8.5% had no comorbidities while 91.5% had at least one comorbidity. Of the 60 CCC categories, 17.5, 22.3, 19.4, 11.0, 6.2, 3.0, and 12.1% had one through ≥7 comorbidities, respectively. Overall, six diseases of the 60 categories were not documented among pulmonology patients while only one disease had no documentation in the GP patients. No gender difference was found in the percent distribution of the number of comorbid conditions. Figure 3 shows the percentage of patients with a number of comorbidities (0 through 7+) by age group combined across genders (excluding the 30s group due to the small sample size among GP patients). Among GP patients, overall 11.0% had 0 and 39.8% had more than 7 comorbidities, and among pulmonologist patients, it was 7.5% and 1.5%, respectively.

### Type of comorbidities by gender and age group differentiated by practice types

For all patients, 12 out of the 60 CCC showed a prevalence greater than 10%, while 15 disease categories had a prevalence between 5 and <10%, and 20 conditions between 1 and <5%. Only 13 comorbidity categories showed less than a 1% prevalence. Table 2 displays the overall frequency of each comorbidity category by gender. Including all patients, the most frequently documented comorbidity among COPD patients was hypertension. Among the 60 CCC with a >10% frequency rate, men were documented significantly more often with other respiratory diseases, sleep disorders, ischemic heart diseases, diabetes, other psychiatric and behavioral diseases, and heart failure than women did. Women had more asthma, allergy, and esophagus, stomach, and duodenum diseases than men did.

**Table 2:** Frequency of chronic comorbid conditions (CCC) by gender.

	Women		Men		All	
Categories	*n* = 3573	%	*n* = 4384	%	*N* = 7957	%
Hypertension	1393	38.99	1758	40.10	3151	39.60
(COPD), emphysema, chronic bronchitis* ^a^	1221	34.17	1594	36.36	2815	35.38
Other respiratory diseases***	823	23.03	1202	27.42	2025	25.45
Sleep disorders***	598	16.74	1322	30.16	1920	24.13
Asthma***	988	27.65	732	16.70	1720	21.62
Ischemic heart disease***	544	15.23	1099	25.07	1643	20.65
Allergy***	678	18.98	501	11.43	1179	14.82
Diabetes***	426	11.92	690	15.74	1116	14.03
Other psychiatric and behavioral diseases***	335	9.38	539	12.29	874	10.98
Esophagus, stomach, and duodenum diseases***	442	12.37	410	9.35	852	10.71
Obesity	392	10.97	451	10.29	843	10.59
Heart failure**	322	9.01	475	10.83	797	10.02
Peripheral neuropathy	379	10.61	410	9.35	789	9.92
Dorsopathies	373	10.44	402	9.17	775	9.74
Other musculoskeletal and joint diseases*	335	9.38	354	8.07	689	8.66
Ear, nose, throat diseases***	319	8.93	287	6.55	606	7.62
Other metabolic diseases	259	7.25	313	7.14	572	7.19
Dyslipidemia	248	6.94	322	7.34	570	7.16
Osteoarthritis and other degenerative joint diseases	258	7.22	274	6.25	532	6.69
Inflammatory arthropathies	226	6.33	293	6.68	519	6.52
Neurotic, stress-related and somatoform diseases***	274	7.67	243	5.54	517	6.50
Depression and mood diseases***	282	7.89	197	4.49	479	6.02
Thyroid diseases***	283	7.92	193	4.40	476	5.98
Chronic infectious diseases	195	5.46	273	6.23	468	5.88
Blood and blood forming organ diseases	206	5.77	217	4.95	423	5.32
Anemia	194	5.43	212	4.84	406	5.10
Cerebrovascular disease	176	4.93	229	5.22	405	5.09
Venous and lymphatic diseases***	222	6.21	174	3.97	396	4.98
Other cardiovascular diseases*	150	4.20	237	5.41	387	4.86
Colitis and related diseases	172	4.81	211	4.81	383	4.81
Peripheral vascular disease**	126	3.53	225	5.13	351	4.41
Atrial fibrillation**	125	3.50	208	4.74	333	4.18
Osteoporosis***	195	5.46	89	2.03	284	3.57
Cataract and other lens diseases	103	2.88	119	2.71	222	2.79
Chronic pancreas, biliary tract and gallbladder diseases**	122	3.41	100	2.28	222	2.79
Deafness, hearing impairment	92	2.57	106	2.42	198	2.49
Other genitourinary diseases**	111	3.11	87	1.98	198	2.49
Other neurological diseases	85	2.38	100	2.28	185	2.32
Chronic kidney diseases	74	2.07	94	2.14	168	2.11
Chronic ulcer of the skin	67	1.88	90	2.05	157	1.97
Cardiac valve diseases	76	2.13	74	1.69	150	1.89
Autoimmune diseases	73	2.04	75	1.71	148	1.86
Dementia	54	1.51	71	1.62	125	1.57
Migraine and facial pain syndromes***	77	2.16	37	0.84	114	1.43
Bradycardias and conduction diseases*	37	1.04	74	1.69	111	1.39
Prostate diseases***	1	0.03	89	2.03	90	1.13
Glaucoma	36	1.01	44	1.00	80	1.01
Parkinson and parkinsonism	28	0.78	45	1.03	73	0.92
Chronic liver diseases	26	0.73	40	0.91	66	0.83
Other eye diseases	34	0.95	32	0.73	66	0.83
Epilepsy	22	0.62	36	0.82	58	0.73
Other digestive diseases	27	0.76	31	0.71	58	0.73
Hermatological neoplasms	19	0.53	38	0.87	57	0.72
Inflammatory bowel diseases	22	0.62	24	0.55	46	0.58
Other skin diseases	26	0.73	20	0.46	46	0.58
Solid neoplasms	14	0.39	12	0.27	26	0.33
Multiple sclerosis	15	0.42	10	0.23	25	0.31
Blindness, visual impairment	6	0.17	9	0.21	15	0.19
Schizophrenia and delusional diseases	5	0.14	5	0.11	10	0.13
Chromosomal abnormalities	0	0	0	0	0	0

Categories are listed in a descending order based on the frequency of each comorbidity in all patients.

*Gender difference significant at *p* < 0.05, **<0.01, ***<0.001 according to *χ*^2^ tests.

^a^(COPD), emphysema, chronic bronchitis category excludes COPD (J44) diagnoses.

**Fig. 4:**
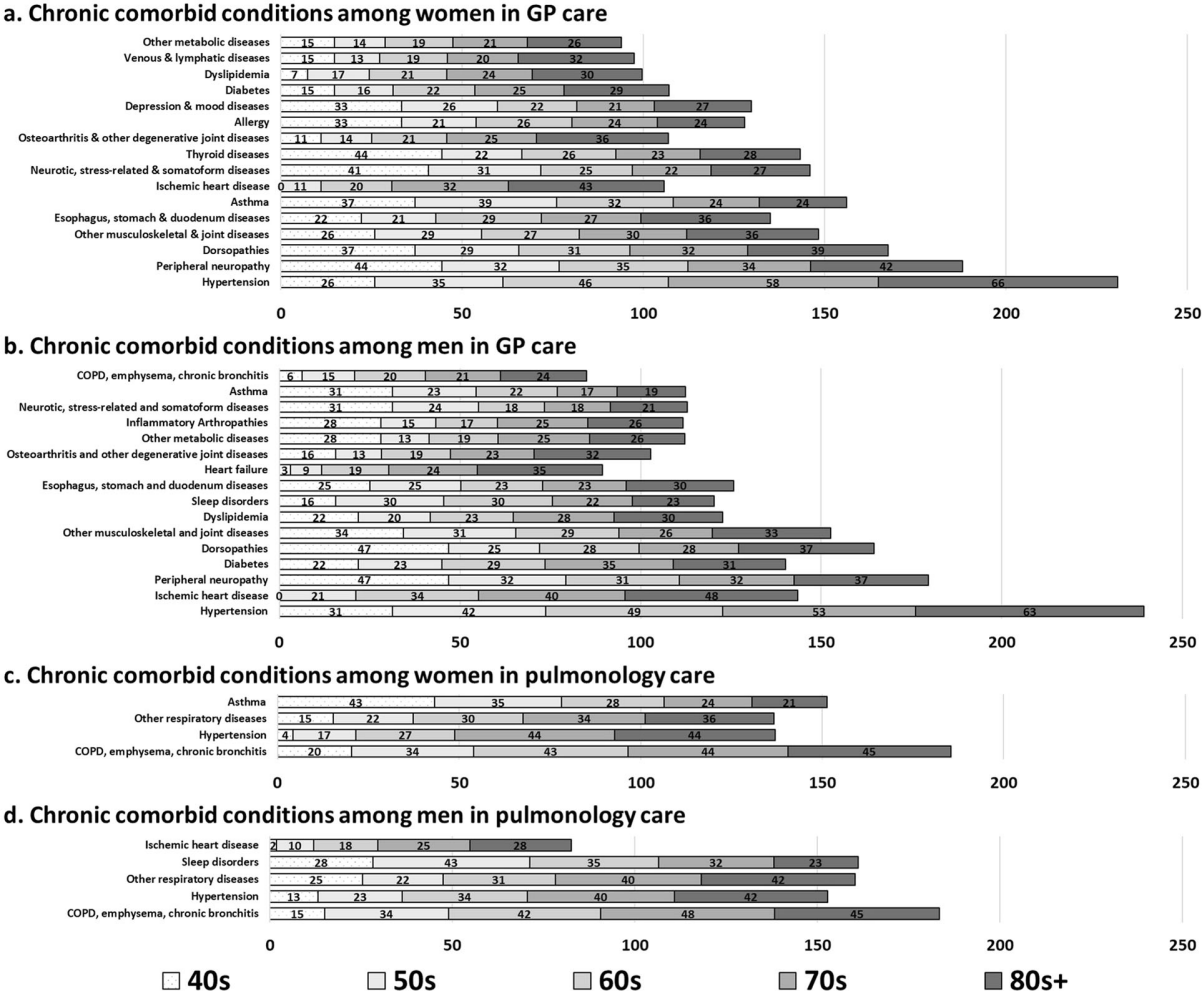
Chronic comorbid conditions (CCC) with >20% frequency by gender and age group for GP and pulmonology practices. Each segment of bars represents the frequency (%) of the listed comorbid condition for each age group. The conditions are presented in the reverse order of overall frequency by gender and practice.

Gender- and age group-specific comorbidities that had a >20% frequency for GP patients and pulmonology patients are presented in Fig. 4. Female patients in GP practice had 30 diseases with a frequency of more than 10% and male patients had 29 diseases (Fig. 4a, b). Sleep and psychological comorbidities were important. Among the top 10 comorbidities, men had sleep disorders (25.1%), and women had neurotic, stress-related, and somatoform diseases (26.1%). Depression and mood diseases were identified in both women (24.1%) and men (13.8%). The frequency of almost all disease categories increased with increasing age for both men and women. However, some diseases were documented more frequently in younger patients compared to older patients such as asthma, thyroid diseases, and neurotic stress-related and somatoform diseases. See Supplementary Document (Supplementary Table 5) for complete data.

In contrast to the GP patients, the pulmonology patients had fewer numbers of comorbidities documented with a frequency greater than 20%: women had four and men had five categories (Fig. 4c, d). In both genders, nine comorbidities were documented with a frequency greater than 10%. Among pulmonology patients, the COPD, emphysema, chronic bronchitis category was most frequently observed in both genders. The frequency of most diseases increased with age.

### Comorbidity during the 10-year COPD index period

After excluding patients who did not have continuous visitation records ±5 years of the first documentation of COPD, 432 GP and 548 pulmonology patients were included in the analysis. Table 3 shows the top 20 chronic comorbidities for the GP and the top 10 for the pulmonology patients by 3-index age groups (age at first COPD documentation <45, 45–64, and ≥65 years old) and over three index periods (Before: >5 years before the first COPD visit; Index Period: ±5 years; and After: >5 years).

**Table 3:** Twenty most frequent comorbidities among GP patients and 10 most frequent comorbidities among pulmonology patients using chronic comorbid conditions (CCC) over the three index periods (5 years before, ±5 years during the index date, and 5 years after).

GP patients comorbidity scores, mean (SD)														
Index age group	Age < 45: *n* = 32				45 ≤ Age < 65: *n* = 218						Age ≥ 65: *n* = 182			
Chronic comorbid conditions	15.13 (8.19)				14.29 (9.09)						16.12 (9.01)			
Elixhauser	5.56 (3.42)				5.96 (4.23)						6.22 (3.84)			
Elixhauser-vW Index	12.81 (10.47)				14.69 (14.13)						15.92 (12.72)			

20 most frequent comorbidities among GP patients (%)														
Disease categories	Total	Before	Index period	After	Disease categories	Total	Before	Index period	After	Disease categories	Total	Before	Index period	After
Dorsopathies	81.3	40.6	31.3	9.4	Hypertension	71.1	18.3	38.5	14.2	Hypertension	81.3	31.9	43.4	6.0
Peripheral neuropathy	78.1	0	31.3	46.9	Peripheral neuropathy	66.5	37.6	23.4	5.5	Peripheral neuropathy	70.9	41.2	24.2	5.5
Neurotic, stress-related & somatoform d.^a^	71.9	25.0	40.6	6.3	Other musculoskeletal & joint d	62.4	27.1	27.1	8.3	Dorsopathies	68.7	43.4	20.3	4.9
Other musculoskeletal & joint d.	68.8	15.6	31.3	21.9	Dorsopathies	61.0	34.9	22.0	4.1	Other musculoskeletal & joint d.	68.7	35.7	28.6	4.4
Esophagus, stomach & duodenum d.	65.6	6.3	50.0	9.4	Esophagus, stomach & duodenum d.	52.8	20.2	26.1	6.4	Osteoarthritis & other degenerative joint d.	62.6	24.7	31.3	6.6
Ear, nose, throat d.	65.6	18.8	37.5	9.4	Neurotic, stress-related & somatoform d.	49.5	21.6	20.6	7.3	Ischemic heart d.	60.4	29.7	24.2	6.6
Depression & mood d.	62.5	9.4	43.8	9.4	Dyslipidemia	47.7	19.7	22.0	6.0	Esophagus, stomach & duodenum d	56.0	19.2	27.5	9.3
Dyslipidemia	59.4	25.0	21.9	12.5	Ischemic heart disease	46.3	17.0	17.9	11.5	Inflammatory Arthropathies	50.0	27.5	19.2	3.3
Thyroid d.	53.1	9.4	28.1	15.6	Osteoarthritis & other degenerative joint d.	44.0	11.9	22.0	10.1	Dyslipidemia	50.0	25.8	22.0	2.2
Other metabolic d.	53.1	21.9	25.0	6.3	Other metabolic d.	43.6	21.6	15.6	6.4	Other metabolic d.	48.9	26.9	20.3	1.6
Asthma	53.1	18.8	18.8	15.6	Thyroid d.	42.7	11.9	21.6	9.2	Heart failure	44.5	12.6	25.8	6.0
Allergy	50.0	18.8	25.0	6.3	Asthma	40.8	7.8	20.6	12.4	Neurotic, stress-related & somatoform d.	44.5	21.4	18.7	4.4
Hypertension	50.0	9.4	21.9	18.8	Diabetes	40.8	15.1	21.1	4.6	Venous & lymphatic d.	42.9	17.0	18.1	7.7
Inflammatory Arthropathies	46.9	21.9	21.9	3.1	Inflammatory Arthropathies	40.8	22.0	16.1	2.8	Diabetes	39.0	14.3	20.3	4.4
Anemia	46.9	25.0	15.6	6.3	Allergy	39.4	13.8	17.4	8.3	Depression & mood d.	39.0	14.3	17.0	7.7
Blood & blood forming organ d.	43.8	9.4	12.5	21.9	Ear, nose, throat d.	38.1	10.6	18.8	8.7	Colitis & related d.	38.5	13.7	14.8	9.9
Osteoarthritis & other degenerative joint d.	43.8	6.3	15.6	21.9	Blood & blood forming organ d.	37.6	5.0	14.2	18.3	Allergy	37.4	12.1	22.5	2.7
Diabetes	40.6	12.5	18.8	9.4	Colitis & related d.	36.2	10.1	15.6	10.6	Cerebrovascular disease	36.3	11.5	16.5	8.2
Colitis & related d.	37.5	6.3	12.5	18.8	Depression & mood d.	35.8	12.4	17.0	6.4	Thyroid d.	36.3	10.4	22.5	3.3
Venous & lymphatic d.	34.4	3.1	12.5	18.8	Venous & lymphatic d.	35.3	9.6	16.5	9.2	Cataract & other lens d.	36.3	6.0	22.0	8.2

Pulmonology patients comorbidity scores, mean (SD)														
Index age group	Age < 45: *n* = 49					45 ≤ Age < 65: *n* = 260					Age ≥ 65: *n* = 239			
Chronic comorbid conditions	3.37 (1.40)					3.18 (1.73)					3.03 (1.53)			
Elixhauser	1.55 (.82)					1.67 (1.14)					1.81 (1.08)			
Elixhauser-vW Index **	3.24 (3.43)					3.68 (3.01)					4.60 (3.4)			

10 most frequent comorbidities among pulmonology patients, %														
Disease categories	Total	Before	Index period	After	Disease categories	Total	Before	Index period	After	Disease categories	Total	Before	Index period	After
Asthma	51.0	30.6	16.3	4.1	(COPD), emphysema, chronic bronchitis	60.8	26.2	28.5	6.2	(COPD), emphysema, chronic bronchitis	61.5	37.2	21.3	2.9
(COPD), emphysema, chronic bronchitis ^b^	49.0	26.5	14.3	8.2	Sleep disorders	36.2	11.5	13.5	11.2	Hypertension	44.4	4.6	22.6	17.2
Allergy	38.8	20.4	10.2	8.2	Other respiratory d.	33.5	9.2	8.1	16.2	Other respiratory d.	38.1	11.7	14.6	11.7
Sleep disorders	32.7	10.2	10.2	12.2	Hypertension	32.7	0.8	15.4	16.5	Sleep disorders	27.2	11.3	10.5	5.4
Other respiratory d.	30.6	22.4	4.10	4.1	Asthma	31.2	21.2	6.9	3.1	Asthma	26.0	18.0	5.9	2.1
Other psychiatric & behavioral d.	24.5	2.0	14.3	8.2	Allergy	17.0	7.7	5	4.2	Ischemic heart disease	21.8	2.5	7.9	11.3
Obesity	22.5	0	4.1	18.4	Obesity	15.0	1.2	5	8.8	Allergy	10.0	7.5	2.1	0.4
Hypertension	20.4	0	6.1	14.3	Other psychiatric & behavioral d	13.1	1.5	9.2	2.3	Diabetes	10.0	0	4.2	5.9
Chronic infectious d.	12.2	0	4.1	8.2	Ischemic heart disease	12.3	0.8	2.7	8.8	Obesity	7.5	1.7	3.8	2.1
Esophagus, stomach, & duodenum d.	8.2	2.0	2.0	4.1	Diabetes	8.1	1.2	1.2	5.8	Other psychiatric & behavioral d.	7.1	0.4	5.4	1.3

**Three index age group difference significant at *p* < 0.01 according to ANOVA.

^a^d.= diseases.

^b^(COPD), emphysema, chronic bronchitis—frequency is based on the data excluding J44 diagnosis.

*GP Patients*. The 3-index age groups of GP patients did not significantly differ by gender (women: <45: 20 (62.5%); 45–64: 89 (40.8%); and ≥65: 84 (46.2%)); however, the mean number of observation years was different among 3-index age groups (<45: 24.4 (6.1) years, 45–64: 22.67 (6.6), and >65: 21.4 (6.3), *p* < 0.05). The <45 index age group had 35 comorbidities with a >10% frequency rate, the 45–64 index age group had 40, and the ≥65 index age group had 41 across three index periods. However, the youngest index age group had a higher number of comorbidities with a >50% frequency than the other two index age groups (13 diseases in the <45, 5 in the 45–64, and 9 in the ≥65 group) (see Table 3). Among the diseases with a >50% frequency rate, in the <45 index age group, esophagus, stomach, and duodenum diseases were documented most frequently during the 10-year index period. They were followed by depression and mood diseases, and neurotic, stress-related and somatoform diseases. Among the 45–64 and ≥65 index age groups, hypertension was the most frequently documented comorbidity during the 10-year index period, and psychological comorbidities diseases did not exceed a 50% frequency rate as did in the younger group.

*Pulmonology Patients*. The 3-index age groups of pulmonology patients did not differ by gender (women: <45: 28 (57.1%); 45-64: 114 (43.8%); and ≥65: 120 (50.2%)). However, as was the case in the GP sample, the mean number of observation years varied within the 3-index age groups (<45: 22.2(4.9) years, 45-65: 22.1 (4.8), ≥65: 20.8 (4.8), *p* < 0.01). The <45 and 45–64 index age groups had nine disease categories with a >10% frequency rate, and ≥65 index age group had eight. Each index age group had only one comorbidity with a >50% frequency; asthma for the youngest index age group, and COPD, emphysema, and chronic bronchitis for the other two groups (see Table 3). Among the comorbidities with a frequency rate greater than 10% during the 10-year index period in pulmonology patients, other psychiatric and behavioral diseases were the only comorbidity that was most often documented during the index period in the <45 index age group. For the 45–64 index age group, (COPD), emphysema, chronic bronchitis, sleep disorders, and other psychiatric and behavioral disorders were most common; and for the >65 index age group, hypertension and other respiratory diseases were most common during the 10-year index period compared to the before or after periods. Other psychiatric and behavioral diseases category was documented more frequently during the 10-year index period than before or after periods combined in all 3-index age groups.

## Discussion

The present study examined gender- and age group-specific chronic comorbidities in primary care patients with COPD stratified by the medical practice type: GP and pulmonology practices. Our main findings were threefold. Firstly, the type of medical practice mattered in documented numbers and types of chronic comorbidities. Secondly, a gender difference was seen in the type of but not in the number of comorbid conditions. Lastly, although increasing age was associated with an increasing number of comorbidities, our younger groups of patients with COPD also displayed multiple comorbidities. Overall, less than 1 in 10 patients had no documented comorbidities, and 75% of the patients had two or more comorbidities.

GPs documented a higher number of and more heterogeneous comorbidities while pulmonologists documented comorbidities more specific to their specialty. This implies that compiled routine data of GP patients display a more complete picture of patients’ health. Our data indicate that in pulmonology practices, diseases affecting other organs or systems other than the lungs are only scarcely documented in the patients’ records. Under- and over-treatment of patients with COPD and comorbidities may result if there is a lack of information and communication about comorbid conditions between these practices and other medical specialists.

Despite having a more complete picture of the patients’ health, primary care physicians report unique challenges of treating COPD due to the medical culture of prioritizing diseases and treating one disease at a time regardless of the reality that most patients suffer from multiple diseases simultaneously^28^. Acute conditions take precedence during unscheduled visits and other conditions such as heart diseases are prioritized during routine visits while the lack of awareness of and concern for COPD further hinders the care of patients with COPD, especially when GPs are pressured for time^28^. Fostering a more holistic approach to medical practice rather than prioritizing and allowing longer consultation durations with the corresponding remuneration can perhaps combat de-prioritization of COPD and enhance early detection of COPD, which could prevent the onset of some of the comorbidities and further improve the treatment of COPD in GP practices.

Examining comorbidities by gender in our study revealed no significant difference in the mean number of comorbidities documented; however, the type of comorbidity was gender-specific, and this difference contributed to the distinct Elixhauser-vW index scores. Men had higher Elixhauser-vW index scores than women due to higher weight points assigned to cardiovascular comorbidities. Although we could not confirm mortality outcomes in our study, previous studies have shown that male COPD patients indeed had higher mortality rates than female COPD patients^12,29^. Men also had more sleep disorders than women did. Considering that poor sleep quality is detrimental to heart health^30^, our study corroborated this link between sleep disorders and cardiovascular diseases in our male patients.

Another important aspect of gender-specific comorbidity was linked to quality of life. Depression and anxiety are some of the most significant predictors of quality of life and health status, even more so than spirometry values in COPD patients^31^. The current study demonstrated, despite having lower Elixhauser-vW index scores than men, women more frequently had chronic diseases that affect mental health, which are known to lower the quality of life^12^. The Elixhauser-vW weight algorithm gives the relative importance of each of the comorbidities as they relate to short-term mortality outcomes^21^. However, with patients reaching old age with multiple comorbid conditions, the quality of life indices or algorithms based on chronic comorbid conditions may be equally important in providing optimal care. To create such an algorithm, investigators must consider gender differences and proactively seek to examine comorbidities that are associated with the health-related quality of life.

Although gender-specific comorbidities belong to discrete categories of diseases, some of them have systemic inflammation as a common denominator. COPD is often accompanied by other inflammatory or inflammation-linked diseases such as skeletal muscular disorder, osteoporosis, obesity, diabetes, cardiovascular diseases, and clinical anxiety and depression^14,32^. Indeed our patient sample displayed a high prevalence of cardiovascular diseases, other musculoskeletal and joint diseases, depression, and diabetes. Concerning the coexistence of this observed disease-clustering, some investigators propose that COPD is at the center of the inflammatory process, while others claim that COPD is one of a multitude of possible manifestations of a chronic systemic inflammation^33,34^. Despite the uncertain mechanism of the relationship, reduction in lung function is a risk factor for arrhythmias, acute coronary events, and cardiovascular disease mortality regardless of smoking status among COPD patients^14^. Furthermore, COPD and the common comorbidities such as cardiovascular diseases, skeletal muscle dysfunction, and osteoporosis contribute to a significantly reduced quality of life. Further advancements in research and the treatment of COPD and its comorbidities are crucial to decipher the relations between COPD and its comorbidities.

With regard to the age-specific comorbidities, older patient groups had more comorbidities than the younger groups. However, the 30s, 40s, and 50s groups also displayed a large number of various comorbidities. It has been suggested that COPD accelerates the aging process^35^. The current study seems to supports this hypothesis, as our findings highlight a multitude of chronic conditions in our younger age groups normally common among older patients. For instance, one in four of our patients in the 40s group had other muscular–skeletal and joint diseases. Moreover, the 10-year COPD index period analyses further corroborated age-specific comorbidities by demonstrating that different comorbidities accompanied COPD during the 10-year index period depending on the age of the first COPD diagnosis. Overall more diseases were found with a higher frequency rate in the <45 index age group compared to the other two index age groups among the GP patients. The results of the present study suggest that physicians must consider and monitor these comorbidities right from the start in the individuals developing COPD at a younger age, since they may interfere with COPD disease management. In a previous study examining difficulties in discussing and treating COPD, primary care physicians reported that if a patient does not fit the typical clinical picture of COPD, this often leads to a lack of diagnosis or treatment^28^. For instance, mild or moderate symptoms of COPD among younger or middle-aged patients tend to be overlooked by physicians because they do not correspond to the assumed clinical picture of COPD. In recent years, multiple phenotypes of COPD have been discussed^36^. Despite this, the heterogeneous nature of COPD may still not be common knowledge among GPs, possibly leading to delayed detection and treatment of COPD among younger patients suffering from the disease.

Similarly, most COPD comorbidity research studies include patients 40 or 45 years and older, while the younger patients are excluded. This may be in part due to difficulties in the accurate differential diagnosis of asthma and COPD^37,38^, and partly because COPD develops slowly and in general becomes apparent in the 40s and 50s^3^. Nevertheless, our small sample of younger patients exhibited different manifestations of COPD when considering its accompanying conditions. They had psychological problems such as depression and anxiety, while older patients had cardiovascular disorders. Although the current study results need to be interpreted with caution due to a small number of younger patients, our findings seem to indicate different clinical phenotypes of COPD among younger patients. Further research focusing on these age groups is warranted to create a detailed classification of the comorbidity in relation to gender, age, and index age at which COPD symptoms occur. Additionally, the different phenotypes need to be further explored to enhance our understanding of COPD and to guide the treatment of choice for effective care.

The strengths of the current investigation include the use of routine real-world data from GP and pulmonology practices, the detailed examination of gender and age group differences, and the use of an extensive number of chronic comorbid disease categories. Previous studies have shown that COPD patients suffered from significant numbers of comorbidities^8,12,39^, and the current study showed gender- and age-specific comorbidities from different practices. Moreover, we examined the comorbidities during the 10-year index period, which provided insights into COPD comorbidity not previously examined. Even one of the most comprehensive data sources such as health insurance claims data normally limit its data handling in the outpatient setting to a maximum of four consecutive years for regulatory reasons^40^. Considering this restriction of the claims data, the comorbidity documented in our study is comprehensive and representative of COPD patients’ long-term health status.

The current study has several weaknesses. First, the use of secondary, partly unstructured routine data resulted in the loss of many incomplete patients’ data. Due to the use of secondary data, the ICD code documentation could not be verified for its completeness and accuracy because examination of an individual routine of documentation in practice was not possible. Second, our study did not include a comparison group. Gender- and age-matched control group could have allowed comorbidity comparisons between patients with and without COPD. However, the aim of this study was to assess and document comorbid conditions by age and gender among patients with COPD in a real-world setting in primary care. Therefore, we investigated the practice-derived routine documentation of COPD and comorbidities for research purposes. Lastly, this study included a relatively small sample size for the younger age groups and thus the interpretation is limited. Similarly, the 10-year index period analysis also had a small sample size. However, 5 years prior and post of the first COPD documentation was deemed an appropriate index period to examine the accompanying medical conditions due to the average progression of COPD. Longitudinal studies with larger sample sizes including longer observation periods and all age groups are warranted.

In conclusion, patients with COPD seeking treatment in primary care settings have a multitude of chronic conditions. Gender- and age-specific comorbid conditions exist and the documented numbers and categories of comorbidities differ depending on the specialty of the medical practice. The lack of awareness of these comorbidities can lead to a treatment delay, low treatment efficacy, and potentially dangerous drug interactions and their side effects. Understanding gender- and age-specific patterns of comorbidity will help to provide the most effective treatment options for all COPD patients. With no cure for COPD presently available, early identification of comorbid conditions and selecting the best treatment options will reduce disease burdens and improve COPD patients’ overall health and quality of life. Physicians treating patients with COPD should be cognizant of these comorbidities, their early onset, and actively assess these conditions. Future studies of COPD comorbidities should include young adult patients and use gender- and age-stratification to make accurate evaluations and implement effective disease and symptom management.

## Supplementary information

Supplementary Figures and Tables

Reporting Summary

## Data Availability

The datasets generated during and/or analyzed during the current study are available from the corresponding author on reasonable request.
