# Supplementary material for: Age- and gender-based comorbidity categories in general practitioner and pulmonology patients with COPD
Source: NPJ Prim Care Respir Med. 2022 May 2;32:17. doi: 10.1038/s41533-022-00278-8 (PMC9061861; doi:10.1038/s41533-022-00278-8)
Supplement: Supplementary file 1 — Supplementary Figures and Tables [file 41533_2022_278_MOESM1_ESM.pdf]

Additional File 1. Supplementary figure and tables

Supplementary Figure 1. Data Flowchart. BeoNet registry patient data for the main and index period analyses

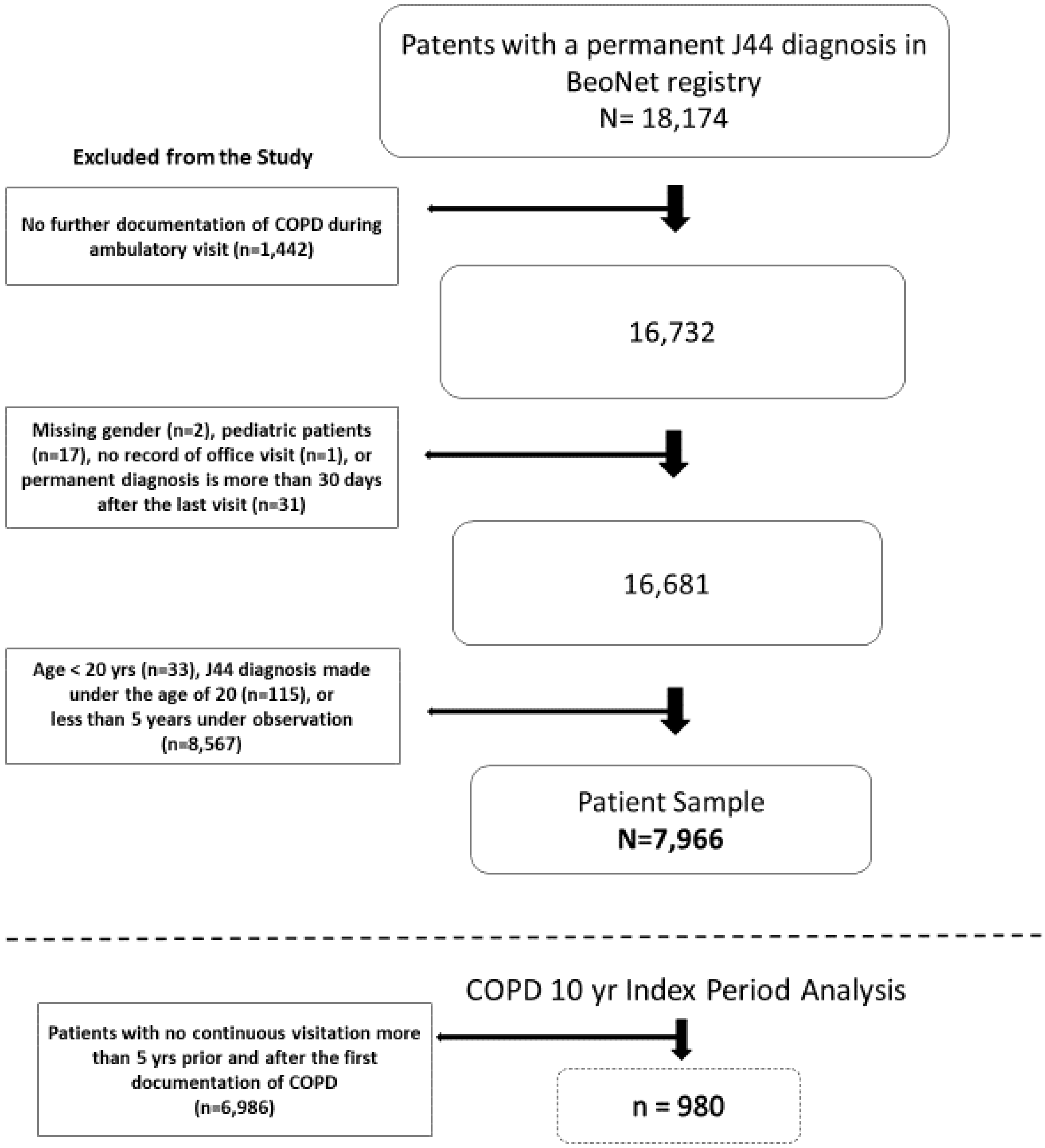

Supplementary Table 1. Post-hoc Bonferroni test p values for the age group comparison

		P values		
**Age Group**	**Comparison Age Group**	**CCC**	**Elixhauser**	**Elixhauser-vW index**
30	40	1.000	1.000	1.000
	50	1.000	1.000	1.000
	60	1.000	0.831	1.000
	70	1.000	0.044	0.126
	80	0.047	0.001	0.000
40	30	1.000	1.000	1.000
	50	1.000	0.899	1.000
	60	0.141	0.000	0.005
	70	0.007	0.000	0.000
	80	0.000	0.000	0.000
50	30	1.000	1.000	1.000
	40	1.000	0.899	1.000
	60	0.256	0.001	0.000
	70	0.001	0.000	0.000
	80	0.000	0.000	0.000
60	30	1.000	0.831	1.000
	40	0.141	0.000	0.005
	50	0.256	0.001	0.000
	70	0.933	0.000	0.001
	80	0.000	0.000	0.000
70	30	1.000	0.044	0.126
	40	0.007	0.000	0.000
	50	0.001	0.000	0.000
	60	0.933	0.000	0.001
	80	0.000	0.000	0.000
80	30	0.047	0.001	0.000
	40	0.000	0.000	0.000
	50	0.000	0.000	0.000
	60	0.000	0.000	0.000
	70	0.000	0.000	0.000

Supplementary Table 2. Elixhauser comorbid conditions by gender and combined

		Women		Men		All	
	vW weight	n = 3573	%	n =4384	%	n = 7957	%
Chronic pulmonary disease (with COPD)	3	3,573	(100.0)	4,384	(100.0)	7,957	(100.0)
Chronic pulmonary disease (no COPD)	3	1,987 ***	(55.6)	2,233	(50.9)	4,220	(53.0)
Hypertension	0	1,393	(39.0)	1,758	(40.1)	3,151	(39.6)
Diabetes, uncomplicated	0	396	(11.1)	635 ***	(14.5)	1,031	(13.0)
Solid tumor without metastasis	4	359	(10.0)	643 ***	(14.7)	1,002	(12.6)
Obesity	-4	392	(11.0)	451	(10.3)	843	(10.6)
Cardiac arrhythmias	5	322	(9.0)	489 **	(11.2)	811	(10.2)
Congestive heart failure	7	282	(7.9)	409 *	(9.3)	691	(8.7)
Depression	-3	291 ***	(8.1)	213	(4.9)	504	(6.3)
Peripheral vascular disorders	2	164	(4.6)	304 ***	(6.9)	468	(5.9)
Pulmonary circulation Disorders	4	154	(4.3)	202	(4.6)	356	(4.5)
Liver disease	11	144	(4.0)	201	(4.6)	345	(4.3)
Rheumatoid arthritis/collagen vascular diseases	0	163 *	(4.6)	160	(3.6)	323	(4.1)
Renal failure	5	119	(3.3)	140	(3.2)	259	(3.3)
Hypothyroidism	0	163 ***	(4.6)	94	(2.1)	257	(3.2)
Diabetes, complicated	0	90	(2.5)	153 *	(3.5)	243	(3.1)
Fluid and electrolyte disorders	5	101	(2.8)	111	(2.5)	212	(2.7)
Deficiency anemia	-2	109 *	(3.1)	100	(2.3)	209	(2.6)
Coagulopathy	3	73	(2.0)	107	(2.4)	180	(2.3)
Other neurological disorders	6	67	(1.9)	106	(2.4)	173	(2.2)
Valvular disease	-1	74	(2.1)	70	(1.6)	144	(1.8)
Weight loss	6	61	(1.7)	67	(1.5)	128	(1.6)
Peptic ulcer disease excluding bleeding	0	49	(1.4)	63	(1.4)	112	(1.4)
Alcohol abuse	0	29	(0.8)	69 **	(1.6)	98	(1.2)
Metastatic Cancer	12	29	(0.8)	57 *	(1.3)	86	(1.1)
Blood loss anemia	-2	24	(0.7)	22	(0.5)	46	(0.6)
Lymphoma	9	13	(0.4)	23	(0.5)	36	(0.5)
Paralysis	7	16	(0.4)	17	(0.4)	33	(0.4)
Psychoses	0	14	(0.4)	13	(0.3)	27	(0.3)
Drug abuse	-7	8	(0.2)	15	(0.3)	23	(0.3)
AIDS/HIV	0	1	(0.0)	2	(0.0)	3	(0.0)

Elixhauser categories are listed in a descending order based on comorbidity frequency of all patients excluding the 20s group.

* Gender difference significant at p < .05; ** p<.01; *** p<.001 according to Chi-squared tests.

Supplementary Table 3. Chronic comorbid conditions (CCC), Elixhauser, and Elixhauser-van Walraven index comorbidity by gender and age for practice types (GPs and Pulmonologists)

	**Chronic comorbid conditions (CCC) †§**																			
	**GP**										**Pulmonology**									
	**Women**					**Men**					**Women**					**Men**				
**Age Group**	n	M	(SD)	Mdn	Range	n	M	(SD)	Mdn	Range	n	M	(SD)	Mdn	Range	n	M	(SD)	Mdn	Range
**30s [a]**	7	7.71	5.56	7	0-19	6	9.83	7.41	8.5	1-20	27	1.85	1.20	2	0-5	34	2.06	1.30	2	0-5
**40s**	27	5.96	4.78	5	0-16	32	6.44	5.87	5	0-19	118	2.06	1.34	2	0-7	106	1.97	1.40	2	0-7
**50s**	136	5.84	6.57	3	0-29	151	5.90	6.20	3	0-32	357	2.18	1.46	2	0-9	433	2.47	1.53	2	0-8
**60s**	285	7.01	7.95	3	0-35	312	6.78	7.46	3	0-31	708	2.45	1.62	2	0-8	843	2.60	1.52	2	0-9
**70s**	271	7.49	8.36	3	0-32	356	7.80	8.57	4	0-36	777	2.69	1.57	3	0-9	1074	2.87	1.65	3	0-9
**80+**	294	10.26	10.25	6	0-42	315	9.51	9.53	6	0-37	566	2.57	1.44	2	0-7	722	2.68	1.59	3	0-9
**Total**	1,020	7.90	8.68			1,172	7.72	8.31			2,553	2.49	1.54			3,212	2.66	1.59		
	**Elixhauser †§**																			
**30s [a]**	7	2.86	2.48	3	1-8	6	3.67	3.39	3.5	0-8	27	.96	.90	1	0-4	34	.91	.71	1	0-3
**40s**	27	2.33	1.78	2	0-6	32	2.72	2.82	2	0-10	118	.90	.79	1	0-4	106	.87	.86	1	0-4
**50s**	136	2.57	2.74	2	0-12	151	2.63	2.78	2	0-13	357	1.04	.83	1	0-5	433	1.19	1.03	1	0-5
**60s**	285	3.09	3.41	2	0-16	312	3.17	3.48	2	0-15	708	1.32	.99	1	0-4	843	1.40	1.05	1	0-6
**70s**	271	3.48	3.70	2	0-18	356	3.66	3.81	2	0-19	777	1.63	1.08	1	0-6	1074	1.67	1.16	1	0-7
**80+**	294	4.15	4.08	3	0-22	315	4.12	4.03	3	0-18	566	1.61	1.10	2	0-7	722	1.70	1.17	2	0-6
**Total**	1,020	3.44	3.67			1,172	3.49	3.67			2,553	1.41	1.04			3,212	1.50	1.13		
	**Elixhauser-van Walraven index *†§**																			
**30s [a]**	7	5.29	5.41	3	0-15	6	13.00	9.06	14.5	3-27	27	2.11	1.69	3	-1-3	34	2.97	1.85	3	-1-10
**40s**	27	3.56	4.59	3	-4-16	32	7.66	8.50	3	-7-36	118	2.88	2.17	3	-4-10	106	2.86	2.09	3	-1-11
**50s**	136	5.80	7.65	3	-4-36	151	6.26	7.42	3	-9-32	357	2.83	2.17	3	-4-19	433	3.23	2.73	3	-4-26
**60s**	285	7.66	9.76	3	-4-60	312	9.47	10.72	3	-4-53	708	3.35	2.84	3	-5-19	843	3.77	2.88	3	-4-19
**70s**	271	9.02	9.67	3	-1-46	356	10.95	11.51	7	-1-55	777	3.87	3.09	3	-4-19	1074	4.55	3.68	3	-4-25
**80+**	294	11.27	11.19	7	-4-67	315	12.08	12.28	7	-1-61	566	4.33	3.28	3	-1-19	722	5.03	3.75	3	-1-24
**Total**	1,020	8.69	10.00			1,172	10.18	11.13			2,553	3.62	2.95			3,212	4.20	3.39	3	

[a] The 30s group was excluded from GPs vs. Pulmonologists analyses due to the small sample size but the raw data are presented here.

* Gender group difference significant at $p < .001$; †Age group difference significant at $p < .001$; § Practice difference significant at $p < .001$ based on ANOVA.

Supplementary Table 4. Post-hoc Bonferroni test p values for the age group comparison by practice types

		**P values**					
		**GP Patients**			**Pulmonology Patients**		
**Age Group**	**Comparison Age Group**	**CCC**	**Elixhauser**	**Elixhauser-vW Index**	**CCC**	**Elixhauser**	**Elixhauser-vW Index**
40	50	1.000	1.000	1.000	0.060	0.031	1.000
	60	1.000	1.000	0.481	0.000	0.000	0.018
	70	1.000	0.355	0.024	0.000	0.000	0.000
	80	0.014	0.013	0.000	0.000	0.000	0.000
50	40	1.000	1.000	1.000	0.060	0.031	1.000
	60	0.924	0.397	0.007	0.051	0.000	0.001
	70	0.027	0.001	0.000	0.000	0.000	0.000
	80	0.000	0.000	0.000	0.000	0.000	0.000
60	40	1.000	1.000	0.481	0.000	0.000	0.018
	50	0.924	0.397	0.007	0.051	0.000	0.001
	70	1.000	0.316	0.118	0.000	0.000	0.000
	80	0.000	0.000	0.000	0.879	0.000	0.000
70	40	1.000	0.355	0.024	0.000	0.000	0.000
	50	0.027	0.001	0.000	0.000	0.000	0.000
	60	1.000	0.316	0.118	0.000	0.000	0.000
	80	0.000	0.072	0.084	0.043	1.000	0.001
80	40	0.014	0.013	0.000	0.000	0.000	0.000
	50	0.000	0.000	0.000	0.000	0.000	0.000
	60	0.000	0.000	0.000	0.879	0.000	0.000
	70	0.000	0.072	0.084	0.043	1.000	0.001

The 30s group was excluded from the analysis due to the small sample size.

Supplementary Table 5. Gender and age stratified comorbidity per practice type.

General Practitioner													
	Women												
**Chronic Comorbid Disease Categories**	**All = 1,013**			**40s = 27**		**50s = 136**		**60s = 285**		**70s = 271**		**80s+ = 294**	
	**n**	**%**		**n**	**%**	**n**	**%**	**n**	**%**	**n**	**%**	**n**	**%**
Hypertension	536	52.9		7	25.9	48	35.3	130	45.6	157	57.9	194	66.0
Peripheral neuropathy	372	36.7		12	44.4	44	32.4	101	35.4	92	33.9	123	41.8
Dorsopathies	338	33.4		10	37.0	39	28.7	87	30.5	88	32.5	114	38.8
Other musculoskeletal & joint diseases	311	30.7		7	25.9	40	29.4	77	27.0	80	29.5	107	36.4
Esophagus, stomach & duodenum diseases	296	29.2		6	22.2	28	20.6	83	29.1	74	27.3	105	35.7
Asthma	290	28.6		10	37.0	53	39.0	92	32.3	64	23.6	71	24.1
Ischemic heart disease	284	28.0		0	0.0	15	11.0	56	19.6	87	32.1	126	42.9
Neurotic, stress-related & somatoform diseases	264	26.1		11	40.7	42	30.9	72	25.3	59	21.8	80	27.2
Thyroid diseases	260	25.7		12	44.4	30	22.1	74	26.0	63	23.2	81	27.6
Osteoarthritis & other degenerative joint diseases	255	25.2		3	11.1	19	14.0	59	20.7	67	24.7	107	36.4
Allergy	247	24.4		9	33.3	28	20.6	75	26.3	64	23.6	71	24.1
Depression & mood diseases	244	24.1		9	33.3	36	26.5	62	21.8	58	21.4	79	26.9
Diabetes	242	23.9		4	14.8	22	16.2	64	22.5	67	24.7	85	28.9
Dyslipidemia	239	23.6		2	7.4	23	16.9	61	21.4	64	23.6	89	30.3
Venous & lymphatic diseases	221	21.8		4	14.8	17	12.5	53	18.6	53	19.6	94	32.0
Other metabolic diseases	208	20.5		4	14.8	19	14.0	53	18.6	56	20.7	76	25.9
Heart failure	202	19.9		1	3.7	9	6.6	34	11.9	59	21.8	99	33.7
Inflammatory Arthropathies	196	19.3		3	11.1	14	10.3	49	17.2	53	19.6	77	26.2
Sleep disorders	192	19.0		4	14.8	28	20.6	65	22.8	50	18.5	45	15.3
Anemia	191	18.9		4	14.8	21	15.4	50	17.5	48	17.7	68	23.1
Ear, nose, throat diseases	189	18.7		7	25.9	31	22.8	44	15.4	49	18.1	58	19.7
(COPD), emphysema, chronic bronchitis [a]	172	17.0		2	7.4	20	14.7	55	19.3	51	18.8	44	15.0
Colitis & related diseases	169	16.7		3	11.1	17	12.5	38	13.3	39	14.4	72	24.5
Cerebrovascular diseases	153	15.1		0	0.0	6	4.4	27	9.5	36	13.3	84	28.6
Blood & blood forming organ diseases	147	14.5		2	7.4	17	12.5	45	15.8	33	12.2	50	17.0
Other cardiovascular diseases	121	11.9		2	7.4	14	10.3	26	9.1	29	10.7	50	17.0
Chronic pancreas, biliary tract & gallbladder diseases	121	11.9		2	7.4	11	8.1	33	11.6	22	8.1	53	18.0
Peripheral vascular diseases	114	11.3		0	0.0	3	2.2	24	8.4	34	12.5	53	18.0
Osteoporosis	112	11.1		1	3.7	2	1.5	19	6.7	30	11.1	60	20.4

Other genitourinary diseases	111	11.0		0	0.0	6	4.4	28	9.8	25	9.2	52	17.7
Cataract and other lens diseases	98	9.7		0	0.0	1	0.7	12	4.2	30	11.1	55	18.7
Deafness, hearing impairment	92	9.1		2	7.4	6	4.4	16	5.6	24	8.9	44	15.0
Atrial Fibrillation	79	7.8		1	3.7	1	0.7	8	2.8	19	7.0	50	17.0
Obesity	77	7.6		3	11.1	11	8.1	26	9.1	25	9.2	12	4.1
Other neurological diseases	76	7.5		3	11.1	3	2.2	20	7.0	16	5.9	34	11.6
Migraine and facial pain syndromes	71	7.0		1	3.7	18	13.2	20	7.0	15	5.5	17	5.8
Chronic ulcer of the skin	67	6.6		0	0.0	4	2.9	10	3.5	9	3.3	44	15.0
Cardiac valve diseases	59	5.8		0	0.0	3	2.2	9	3.2	16	5.9	31	10.5
Dementia	53	5.2		0	0.0	0	0.0	2	0.7	8	3.0	43	14.6
Chronic kidney diseases	57	5.6		0	0.0	3	2.2	11	3.9	14	5.2	29	9.9
Other psychiatric and behavioral diseases	68	6.7		2	7.4	9	6.6	22	7.7	16	5.9	19	6.5
Other respiratory diseases	48	4.7		2	7.4	9	6.6	14	4.9	8	3.0	15	5.1
Chronic infectious diseases	46	4.5		2	7.4	4	2.9	15	5.3	10	3.7	15	5.1
Epilepsy	15	1.5		2	7.4	1	0.7	1	0.4	4	1.5	7	2.4
Autoimmune diseases	53	5.2		1	3.7	8	5.9	17	6.0	7	2.6	20	6.8
Other eye diseases	33	3.3		0	0.0	2	1.5	9	3.2	6	2.2	16	5.4
Other digestive diseases	27	2.7		0	0.0	2	1.5	3	1.1	6	2.2	16	5.4
Bradycardias and conduction diseases	25	2.5		0	0.0	2	1.5	2	0.7	5	1.8	16	5.4
Parkinson and parkinsonism	24	2.4		0	0.0	0	0.0	2	0.7	10	3.7	12	4.1
Glaucoma	22	2.2		0	0.0	0	0.0	3	1.1	7	2.6	12	4.1
Other skin diseases	25	2.5		1	3.7	0	0.0	9	3.2	5	1.8	10	3.4
Chronic liver diseases	25	2.5		0	0.0	1	0.7	10	3.5	4	1.5	10	3.4
Solid neoplasms	14	1.4		0	0.0	0	0.0	5	1.8	1	0.4	8	2.7
Hermatological neoplasms	13	1.3		0	0.0	0	0.0	3	1.1	7	2.6	3	1.0
Inflammatory bowel diseases	17	1.7		0	0.0	2	1.5	6	2.1	4	1.5	5	1.7
Multiple sclerosis	6	0.6		0	0.0	2	1.5	3	1.1	0	0.0	1	0.3
Blindness, visual impairment	6	0.6		0	0.0	0	0.0	1	0.4	1	0.4	4	1.4
Schizophrenia and delusional diseases	5	0.5		0	0.0	0	0.0	2	0.7	2	0.7	1	0.3
Prostate diseases	1	0.1		0	0.0	0	0.0	0	0.0	0	0.0	1	0.3
Chromosomal abnormalities	0	0											
	**Men**												
	**All = 1,166**			**40s = 32**		**50s = 151**		**60s = 312**		**70s = 356**		**80s+ = 315**	
Hypertension	616	52.8		10	31.3	64	42.4	153	49.0	190	53.4	199	63.2

Ischemic heart disease	433	37.1		0	0.0	32	21.2	106	34.0	144	40.4	151	47.9
Peripheral neuropathy	392	33.6		15	46.9	49	32.5	98	31.4	113	31.7	117	37.1
Diabetes	355	30.4		7	21.9	35	23.2	89	28.5	126	35.4	98	31.1
Dorsopathies	355	30.4		15	46.9	38	25.2	86	27.6	98	27.5	118	37.5
Other musculoskeletal and joint diseases	342	29.3		11	34.4	47	31.1	89	28.5	92	25.8	103	32.7
Dyslipidemia	303	26.0		7	21.9	30	19.9	72	23.1	99	27.8	95	30.2
Sleep disorders	294	25.2		5	15.6	45	29.8	94	30.1	79	22.2	71	22.5
Esophagus, stomach and duodenum diseases	293	25.1		8	25.0	38	25.2	71	22.8	82	23.0	94	29.8
Heart failure	268	23.0		1	3.1	13	8.6	58	18.6	87	24.4	109	34.6
Osteoarthritis and other degenerative joint diseases	268	23.0		5	15.6	19	12.6	60	19.2	82	23.0	102	32.4
Other metabolic diseases	262	22.5		9	28.1	20	13.2	60	19.2	90	25.3	83	26.3
Inflammatory Arthropathies	257	22.0		9	28.1	23	15.2	53	17.0	89	25.0	83	26.3
Neurotic, stress-related and somatoform diseases	235	20.2		10	31.3	36	23.8	57	18.3	65	18.3	67	21.3
Asthma	234	20.1		10	31.3	35	23.2	70	22.4	59	16.6	60	19.0
(COPD), emphysema, chronic bronchitis	234	20.1		2	6.3	22	14.6	61	19.6	74	20.8	75	23.8
Colitis and related diseases	210	18.0		6	18.8	19	12.6	51	16.3	63	17.7	71	22.5
Anemia	207	17.8		7	21.9	19	12.6	42	13.5	67	18.8	72	22.9
Cerebrovascular disease	202	17.3		1	3.1	12	7.9	32	10.3	76	21.3	81	25.7
Allergy	190	16.3		7	21.9	26	17.2	54	17.3	50	14.0	53	16.8
Thyroid diseases	185	15.9		8	25.0	24	15.9	48	15.4	51	14.3	54	17.1
Peripheral vascular disease	182	15.6		2	6.3	10	6.6	38	12.2	78	21.9	54	17.1
Venous and lymphatic diseases	172	14.8		1	3.1	12	7.9	40	12.8	50	14.0	69	21.9
Other cardiovascular diseases	168	14.4		2	6.3	12	7.9	30	9.6	68	19.1	56	17.8
Ear, nose, throat diseases	165	14.2		10	31.3	22	14.6	35	11.2	44	12.4	54	17.1
Other psychiatric and behavioral diseases	164	14.1		3	9.4	23	15.2	53	17.0	52	14.6	33	10.5
Depression and mood diseases	161	13.8		7	21.9	26	17.2	38	12.2	43	12.1	47	14.9
Blood and blood forming organ diseases	160	13.7		3	9.4	18	11.9	36	11.5	47	13.2	56	17.8
Atrial Fibrillation	129	11.1		1	3.1	4	2.6	32	10.3	41	11.5	51	16.2
Cataract and other lens diseases	107	9.2		0	0.0	1	0.7	10	3.2	35	9.8	61	19.4
Deafness, hearing impairment	103	8.8		1	3.1	3	2.0	24	7.7	25	7.0	50	15.9
Chronic pancreas, biliary tract and gallbladder diseases	98	8.4		5	15.6	10	6.6	18	5.8	27	7.6	38	12.1
Obesity	95	8.1		4	12.5	12	7.9	27	8.7	31	8.7	21	6.7
Chronic ulcer of the skin	90	7.7		0	0.0	7	4.6	15	4.8	29	8.1	39	12.4
Prostate diseases	89	7.6		1	3.1	5	3.3	10	3.2	31	8.7	42	13.3

Other genitourinary diseases	86	7.4		3	9.4	4	2.6	15	4.8	31	8.7	33	10.5
Other neurological diseases	85	7.3		1	3.1	6	4.0	14	4.5	25	7.0	39	12.4
Other respiratory diseases	83	7.1		0	0.0	9	6.0	19	6.1	25	7.0	30	9.5
Dementia	67	5.7		0	0.0	1	0.7	3	1.0	16	4.5	47	14.9
Chronic infectious diseases	64	5.5		0	0.0	7	4.6	19	6.1	18	5.1	20	6.3
Chronic kidney diseases	64	5.5		2	6.3	2	1.3	12	3.8	21	5.9	27	8.6
Autoimmune diseases	62	5.3		0	0.0	5	3.3	17	5.4	21	5.9	19	6.0
Cardiac valve diseases	51	4.4		0	0.0	8	5.3	6	1.9	13	3.7	24	7.6
Bradycardias and conduction diseases	46	3.9		0	0.0	1	0.7	8	2.6	18	5.1	19	6.0
Osteoporosis	45	3.9		0	0.0	1	0.7	10	3.2	18	5.1	16	5.1
Chronic liver diseases	36	3.1		1	3.1	9	6.0	15	4.8	9	2.5	2	0.6
Glaucoma	35	3.0		1	3.1	3	2.0	7	2.2	9	2.5	15	4.8
Migraine and facial pain syndromes	34	2.9		1	3.1	7	4.6	12	3.8	8	2.2	6	1.9
Other digestive diseases	31	2.7		1	3.1	1	0.7	8	2.6	8	2.2	13	4.1
Other eye diseases	31	2.7		1	3.1	2	1.3	8	2.6	8	2.2	12	3.8
Parkinson and parkinsonism	27	2.3		0	0.0	1	0.7	1	0.3	12	3.4	13	4.1
Epilepsy	23	2.0		0	0.0	6	4.0	3	1.0	9	2.5	5	1.6
Hermatological neoplasms	23	2.0		2	6.3	2	1.3	4	1.3	8	2.2	7	2.2
Inflammatory bowel diseases	21	1.8		0	0.0	2	1.3	6	1.9	7	2.0	6	1.9
Other skin diseases	20	1.7		0	0.0	0	0.0	9	2.9	6	1.7	5	1.6
Solid neoplasms	10	0.9		0	0.0	0	0.0	1	0.3	4	1.1	5	1.6
Blindness, visual impairment	9	0.8		0	0.0	1	0.7	0	0.0	4	1.1	4	1.3
Multiple sclerosis	9	0.8		0	0.0	1	0.7	6	1.9	1	0.3	1	0.3
Schizophrenia and delusional diseases	4	0.3		0	0.0	1	0.7	1	0.3	0	0.0	2	0.6
Chromosomal abnormalities	0	0											
**Pulmonologist**													
	**Women**												
	**All = 2,526**			**40s = 118**		**50s = 357**		**60s = 708**		**70s = 777**		**80s+ = 566**	
(COPD), emphysema, chronic bronchitis	1,042	41.3		24	20.3	120	33.6	302	42.7	342	44.0	254	44.9
Hypertension	852	33.7		5	4.2	62	17.4	193	27.3	341	43.9	251	44.3
Other respiratory diseases	773	30.6		18	15.3	79	22.1	214	30.2	261	33.6	201	35.5
Asthma	681	27.0		51	43.2	125	35.0	200	28.2	188	24.2	117	20.7
Allergy	420	16.6		44	37.3	92	25.8	129	18.2	100	12.9	55	9.7

Sleep disorders	404	16.0		15	12.7	60	16.8	143	20.2	123	15.8	63	11.1
Obesity	308	12.2		16	13.6	46	12.9	92	13.0	102	13.1	52	9.2
Other psychiatric and behavioral diseases	261	10.3		20	16.9	59	16.5	99	14.0	68	8.8	15	2.7
Ischemic heart disease	260	10.3		0	0.0	9	2.5	41	5.8	107	13.8	103	18.2
Diabetes	182	7.2		1	0.8	13	3.6	31	4.4	81	10.4	56	9.9
Chronic infectious diseases	147	5.8		7	5.9	22	6.2	39	5.5	52	6.7	27	4.8
Esophagus, stomach and duodenum diseases	142	5.6		5	4.2	12	3.4	44	6.2	48	6.2	33	5.8
Ear, nose, throat diseases	127	5.0		8	6.8	18	5.0	35	4.9	38	4.9	28	4.9
Heart failure	120	4.8		2	1.7	4	1.1	20	2.8	45	5.8	49	8.7
Osteoporosis	83	3.3		1	0.8	4	1.1	13	1.8	38	4.9	27	4.8
Blood and blood forming organ diseases	58	2.3		4	3.4	8	2.2	11	1.6	16	2.1	19	3.4
Other metabolic diseases	50	2.0		5	4.2	10	2.8	27	3.8	7	0.9	1	0.2
Atrial Fibrillation	46	1.8		0	0.0	0	0.0	4	0.6	16	2.1	26	4.6
Depression and mood diseases	36	1.4		4	3.4	6	1.7	15	2.1	7	0.9	4	0.7
Dorsopathies	32	1.3		0	0.0	5	1.4	10	1.4	9	1.2	8	1.4
Inflammatory Arthropathies	29	1.1		1	0.8	2	0.6	12	1.7	9	1.2	5	0.9
Other cardiovascular diseases	28	1.1		0	0.0	1	0.3	2	0.3	14	1.8	11	1.9
Cerebrovascular disease	23	0.9		1	0.8	2	0.6	4	0.6	9	1.2	7	1.2
Thyroid diseases	22	0.9		0	0.0	5	1.4	6	0.8	7	0.9	4	0.7
Autoimmune diseases	20	0.8		0	0.0	2	0.6	6	0.8	8	1.0	4	0.7
Other musculoskeletal and joint diseases	18	0.7		0	0.0	3	0.8	6	0.8	9	1.2	0	0.0
Chronic kidney diseases	17	0.7		0	0.0	1	0.3	3	0.4	7	0.9	6	1.1
Cardiac valve diseases	16	0.6		0	0.0	0	0.0	5	0.7	6	0.8	5	0.9
Glaucoma	14	0.6		1	0.8	1	0.3	3	0.4	4	0.5	5	0.9
Peripheral vascular disease	12	0.5		0	0.0	0	0.0	4	0.6	5	0.6	3	0.5
Bradycardias and conduction diseases	10	0.4		0	0.0	0	0.0	2	0.3	3	0.4	5	0.9
Multiple sclerosis	9	0.4		2	1.7	1	0.3	5	0.7	1	0.1	0	0.0
Other neurological diseases	9	0.4		1	0.8	1	0.3	4	0.6	1	0.1	2	0.4
Dyslipidemia	8	0.3		0	0.0	2	0.6	0	0.0	6	0.8	0	0.0
Epilepsy	7	0.3		2	1.7	1	0.3	1	0.1	3	0.4	0	0.0
Hermatological neoplasms	6	0.2		0	0.0	0	0.0	1	0.1	4	0.5	1	0.2
Inflammatory bowel diseases	5	0.2		0	0.0	2	0.6	2	0.3	1	0.1	0	0.0
Neurotic, stress-related and somatoform diseases	5	0.2		2	1.7	1	0.3	2	0.3	0	0.0	0	0.0
Cataract and other lens diseases	5	0.2		0	0.0	0	0.0	0	0.0	3	0.4	2	0.4

Migraine and facial pain syndromes	4	0.2		1	0.8	0	0.0	2	0.3	1	0.1	0	0.0
Parkinson and parkinsonism	4	0.2		0	0.0	0	0.0	1	0.1	1	0.1	2	0.4
Peripheral neuropathy	4	0.2		1	0.8	0	0.0	0	0.0	0	0.0	3	0.5
Colitis and related diseases	2	0.1		0	0.0	1	0.3	0	0.0	0	0.0	1	0.2
Anemia	2	0.1		0	0.0	0	0.0	0	0.0	2	0.3	0	0.0
Osteoarthritis and other degenerative joint diseases	2	0.1		1	0.8	0	0.0	1	0.1	0	0.0	0	0.0
Chronic liver diseases	1	0.0		0	0.0	0	0.0	1	0.1	0	0.0	0	0.0
Dementia	1	0.0		0	0.0	0	0.0	1	0.1	0	0.0	0	0.0
Other eye diseases	1	0.0		0	0.0	0	0.0	1	0.1	0	0.0	0	0.0
Blindness, visual impairment	0	0											
Chromosomal abnormalities	0	0											
Chronic pancreas, biliary tract and gallbladder diseases	0	0											
Chronic ulcer of the skin	0	0											
Deafness, hearing impairment	0	0											
Other digestive diseases	0	0											
Other genitourinary diseases	0	0											
Other skin diseases	0	0											
Prostate diseases	0	0											
Schizophrenia and delusional diseases	0	0											
Solid neoplasms	0	0											
Venous and lymphatic diseases	0	0											
	**Men**												
	**All = 3,178**			**40s = 106**		**50s = 433**		**60s = 843**		**70s = 1,074**		**80s+ = 722**	
(COPD), emphysema, chronic bronchitis	1,351	42.5		16	15.1	147	33.9	351	41.6	512	47.7	325	45.0
Hypertension	1,139	35.8		14	13.2	100	23.1	289	34.3	433	40.3	303	42.0
Other respiratory diseases	1,116	35.1		27	25.5	95	21.9	260	30.8	430	40.0	304	42.1
Sleep disorders	1,019	32.1		30	28.3	186	43.0	297	35.2	339	31.6	167	23.1
Ischemic heart disease	665	20.9		2	1.9	44	10.2	148	17.6	271	25.2	200	27.7
Asthma	476	15.0		34	32.1	97	22.4	138	16.4	138	12.8	69	9.6
Other psychiatric and behavioral diseases	367	11.5		15	14.2	94	21.7	123	14.6	109	10.1	26	3.6
Obesity	353	11.1		14	13.2	61	14.1	99	11.7	119	11.1	60	8.3
Diabetes	331	10.4		3	2.8	27	6.2	79	9.4	136	12.7	86	11.9
Allergy	299	9.4		22	20.8	59	13.6	81	9.6	92	8.6	45	6.2
Chronic infectious diseases	208	6.5		4	3.8	26	6.0	48	5.7	82	7.6	48	6.6

Heart failure	205	6.5		2	1.9	10	2.3	39	4.6	85	7.9	69	9.6
Ear, nose, throat diseases	117	3.7		5	4.7	17	3.9	27	3.2	40	3.7	28	3.9
Esophagus, stomach and duodenum diseases	114	3.6		6	5.7	19	4.4	32	3.8	33	3.1	24	3.3
Atrial Fibrillation	78	2.5		0	0.0	5	1.2	13	1.5	24	2.2	36	5.0
Other cardiovascular diseases	69	2.2		0	0.0	5	1.2	13	1.5	34	3.2	17	2.4
Blood and blood forming organ diseases	54	1.7		2	1.9	6	1.4	24	2.8	16	1.5	6	0.8
Other metabolic diseases	48	1.5		4	3.8	10	2.3	15	1.8	11	1.0	8	1.1
Dorsopathies	43	1.4		0	0.0	3	0.7	10	1.2	22	2.0	8	1.1
Peripheral vascular disease	43	1.4		0	0.0	3	0.7	14	1.7	18	1.7	8	1.1
Osteoporosis	43	1.4		1	0.9	5	1.2	12	1.4	14	1.3	11	1.5
Depression and mood diseases	35	1.1		3	2.8	16	3.7	4	0.5	10	0.9	2	0.3
Inflammatory Arthropathies	33	1.0		0	0.0	8	1.8	8	0.9	13	1.2	4	0.6
Chronic kidney diseases	30	0.9		0	0.0	4	0.9	2	0.2	9	0.8	15	2.1
Bradycardias and conduction diseases	28	0.9		1	0.9	1	0.2	2	0.2	11	1.0	13	1.8
Cerebrovascular disease	27	0.8		0	0.0	2	0.5	8	0.9	11	1.0	6	0.8
Cardiac valve diseases	23	0.7		1	0.9	0	0.0	3	0.4	10	0.9	9	1.2
Parkinson and parkinsonism	18	0.6		0	0.0	0	0.0	3	0.4	10	0.9	5	0.7
Dyslipidemia	15	0.5		0	0.0	4	0.9	8	0.9	2	0.2	1	0.1
Peripheral neuropathy	15	0.5		0	0.0	4	0.9	5	0.6	3	0.3	3	0.4
Hermatological neoplasms	15	0.5		0	0.0	1	0.2	1	0.1	8	0.7	5	0.7
Other neurological diseases	13	0.4		1	0.9	3	0.7	2	0.2	6	0.6	1	0.1
Autoimmune diseases	13	0.4		0	0.0	3	0.7	4	0.5	3	0.3	3	0.4
Epilepsy	12	0.4		0	0.0	4	0.9	4	0.5	2	0.2	2	0.3
Cataract and other lens diseases	12	0.4		0	0.0	0	0.0	5	0.6	4	0.4	3	0.4
Other musculoskeletal and joint diseases	10	0.3		2	1.9	0	0.0	3	0.4	3	0.3	2	0.3
Glaucoma	9	0.3		0	0.0	0	0.0	3	0.4	3	0.3	3	0.4
Neurotic, stress-related and somatoform diseases	5	0.2		0	0.0	0	0.0	1	0.1	3	0.3	1	0.1
Thyroid diseases	5	0.2		0	0.0	0	0.0	2	0.2	2	0.2	1	0.1
Dementia	4	0.1		0	0.0	0	0.0	0	0.0	0	0.0	4	0.6
Chronic liver diseases	4	0.1		0	0.0	1	0.2	1	0.1	2	0.2	0	0.0
Osteoarthritis and other degenerative joint diseases	4	0.1		0	0.0	0	0.0	1	0.1	2	0.2	1	0.1
Deafness, hearing impairment	3	0.1		0	0.0	0	0.0	0	0.0	0	0.0	3	0.4
Inflammatory bowel diseases	3	0.1		0	0.0	0	0.0	3	0.4	0	0.0	0	0.0
Migraine and facial pain syndromes	2	0.1		0	0.0	0	0.0	2	0.2	0	0.0	0	0.0

Anemia	2	0.1		0	0.0	0	0.0	0	0.0	2	0.2	0	0.0
Solid neoplasms	2	0.1		0	0.0	0	0.0	0	0.0	1	0.1	1	0.1
Chronic pancreas, biliary tract and gallbladder diseases	1	0.0		0	0.0	0	0.0	1	0.1	0	0.0	0	0.0
Multiple sclerosis	1	0.0		0	0.0	0	0.0	1	0.1	0	0.0	0	0.0
Other genitourinary diseases	1	0.0		0	0.0	0	0.0	1	0.1	0	0.0	0	0.0
Schizophrenia and delusional diseases	1	0.0		0	0.0	0	0.0	1	0.1	0	0.0	0	0.0
Venous and lymphatic diseases	1	0.0		0	0.0	0	0.0	0	0.0	1	0.1	0	0.0
Blindness, visual impairment	0	0											
Chromosomal abnormalities	0	0											
Chronic ulcer of the skin	0	0											
Colitis and related diseases	0	0											
Other digestive diseases	0	0											
Other eye diseases	0	0											
Other skin diseases	0	0											
Prostate diseases	0	0											

[a] (COPD), emphysema, chronic bronchitis excludes J44 diagnosis
